# SUMA: a lightweight machine learning model-powered shared nearest neighbour-based clustering application interface for scRNA-Seq data

**DOI:** 10.55730/1300-0152.2675

**Published:** 2023-12-18

**Authors:** Hamza Umut KARAKURT, Pınar PİR

**Affiliations:** 1 Department of Bioengineering, Faculty of Engineering, Gebze Technical University, Kocaeli, Turkiye; 2 Idea Technology Solutions R&D Center, İstanbul, Turkiye

**Keywords:** ScRNA-Seq, machine learning, clustering, RShiny, random forest

## Abstract

**Background/aim:**

Single-cell transcriptomics (scRNA-Seq) explores cellular diversity at the gene expression level. Due to the inherent sparsity and noise in scRNA-Seq data and the uncertainty on the types of sequenced cells, effective clustering and cell type annotation are essential. The graph-based clustering of scRNA-Seq data is a simple yet powerful approach that presents data as a “shared nearest neighbour” graph and clusters the cells using graph clustering algorithms. These algorithms are dependent on several user-defined parameters.

Here we present SUMA, a lightweight tool that uses a random forest model to predict the optimum number of neighbours to obtain the optimum clustering results. Moreover, we integrated our method with other commonly used methods in an RShiny application. SUMA can be used in a local environment (https://github.com/hkarakurt8742/SUMA) or as a browser tool (https://hkarakurt.shinyapps.io/suma/).

**Materials and methods:**

Publicly available scRNA-Seq datasets and 3 different graph-based clustering algorithms were used to develop SUMA, and a large range for number of neighbours and variant genes was taken into consideration. The quality of clustering was assessed using the adjusted Rand index (ARI) and true labels of each dataset. The data were split into training and test datasets, and the model was built and optimised using Scikit-learn (Python) and randomForest (R) libraries.

**Results:**

The accuracy of our machine learning model was 0.96, while the AUC of the ROC curve was 0.98. The model indicated that the number of cells in scRNA-Seq data is the most important feature when deciding the number of neighbours.

**Conclusion:**

We developed and evaluated the SUMA model and implemented the method in the SUMAShiny app, which integrates SUMA with different clustering methods and enables nonbioinformatician users to cluster and visualise their scRNA data easily. The SUMAShiny app is available both for desktop and browser use.

## 1. Introduction

Single-cell genomics technologies are relatively new yet powerful approaches that enable researchers to investigate biological features at the cellular level. As opposed to tissue-based (bulk) experimental procedures that produce average measurements for the whole sample, single-cell omics technologies, including single-cell genomics (Gawad et al., 2016; Cuomo et al., 2023), transcriptomics (Jovic et al., 2022), epigenomics (Clark et al., 2016), and proteomics (Darmanis and Slavov, 2023), provide data at a single-cell resolution to resolve cellular heterogeneity and reveal rare cell types.

Single-cell RNA sequencing (scRNA-Seq) is a widely used transcriptomics sequencing method that can sequence the mRNA profile of up to millions of single cells (Haque et al., 2017; Chen et al., 2019). The number of cells that can be sequenced using scRNA-Seq increased exponentially from its first use (Tang et al., 2009); since then, various scRNA-Seq experimental procedures have been developed by researchers (Haque et al., 2017). scRNA-Seq experiments often use unique molecular identifiers (UMIs) (Kou et al., 2016) or External RNA Controls Consortium (ERCC) spike-in controls. Both methods serve several important purposes such as quality control, normalisation, and detection of bias.

Independent of the experimental method used, scRNA-Seq data are noisier and sparser than bulk RNA-Seq data. Since an average single cell expresses only a small number of genes (Orphanides and Reinberg, 2002; Shalek et al., 2013), the gene expression matrix (aka count matrix) has a large number of zero values, leading to a sparse dataset (Mukherjee et al., 2018; Jiang et al., 2022). The sparsity of the data makes the traditional data analysis methods and most of the bulk RNA-Seq analysis methods inefficient in scRNA-Seq datasets. Due to the very low amount of RNA that can be extracted from a single cell, the technical noise in the data is high. Noise levels in data can be elevated even more by various reasons such as amplification biases, dropout events, and experiment-to-experiment variability (Janssen et al., 2023). This noisy characteristic of the data obligated researchers to develop noise reduction methods (Chu et al., 2022).

Similar to the quality control and normalisation steps, clustering of scRNA-Seq data also requires more sophisticated methods than the methods used for bulk RNA-Seq (Duò et al., 2020; Yu et al., 2022; Zhang et al., 2023). These methods include adaptations of more traditional methods such as graph-based clustering (Traag et al., 2019; Hloch et al., 2022) or deep-learning-based methods (Tian et al., 2019; Lee et al., 2023) including large language models (LLMs) (Yang et al., 2022).

Graph-based clustering methods (Thomas et al., 2016) are used to reveal the clusters and communities in network structures. These methods are widely used in network biology (Pavlopoulos et al., 2011; Ali et al., 2023). In the field of scRNA-Seq data analysis, graph-based clustering methods are preferable due to their fast and accurate results as opposed to more conventional methods such as hierarchical clustering. Although the clustering power and efficiency of these methods are increasing exponentially, the use of these models still requires high computational power and a certain level of expertise that makes these methods inaccessible for users with noncomputational backgrounds and limited resources.

The first step of graph-based clustering of scRNA-Seq is representing the dataset itself or a reduced subset of it, produced by a dimension reduction algorithm such as principal component analysis (PCA), as a graph. For this purpose, shared nearest neighbour (SNN) is the most common approach. In this approach, each cell is connected to a user selected number of neighbours to build the k-nearest neighbour (kNN) graph. This kNN graph is then used to construct an SNN graph by calculating the neighbourhood overlap using the Jaccard index. In the last step, the constructed graph is clustered using a graph-based clustering algorithm. The quality of the clustering can be measured using various metrics such as the Dunn index (Dunn, 1973) and the silhouette index (Dudek, 2020). The most widely used all-in-one libraries of scRNA-Seq data analysis, Seurat (Hao et al., 2021) and Scran (Lun et al., 2016), use graph-based community detection algorithms. Seurat uses an implementation of Louvain (De Meo et al., 2011), while Scran enables users to use different algorithms such as Walktrap (Pons and Latapy, 2006) and Louvain via the igraph package (Csardi and Nepusz, 2006).

Here we present SUMA, a lightweight, random forest classifier model that predicts the optimum number of neighbours and the optimum community detection algorithm (Walktrap, Leiden, or Louvain) for clustering of a given dataset, using number of cells, number of principal components, number of highly variable genes used in the PCA, experiment type, and percentage of variance represented by the PCA to construct the SNN graph. SUMA is trained using the publicly available Zhengmix data (Zheng et al., 2017) and the Tabula Sapiens (Jones et al., 2022) scRNA-Seq datasets. SUMA is available as a standalone Python terminal application and also as an RShiny application, SUMAShiny, an operating system-free application that uses SUMA itself along with Seurat clustering, Dunn index-optimised Louvain/Leiden-based clustering, and consensus clustering. SUMA and the SUMAShiny are available as desktop applications at github.com/hkarakurt8742/SUMA and SUMAShiny is also available as a browser application at https://hkarakurt.shinyapps.io/suma/.

## 2. Materials and methods

### 2.1. Preprocessing of scRNA-Seq data

Zhengmix datasets (Zhengmix4eq, Zhengmix8eq, and Zhengmix4uneq) were downloaded using the DuoClustering Bioconductor library (Duò et al., 2020). Tabula Sapiens datasets were downloaded from ExperimentHub (Morgan, 2023). The Scran (Lun et al., 2016) package was used, scripts in *the Orchestrating single-cell analysis with Bioconductor* (Amezquita et al., 2020) manual were adapted for custom use, and the count matrix and known cell labels were used as inputs to the analysis pipeline. The datasets were grouped as “UMI” and “Spike” as the preprocessing of these groups requires different filtration approaches. The UMI datasets were filtered based on mitochondrial gene expression, and total counts in each cell and total features in each cell using the mean absolute deviation (MAD) method using the *isOutlier* function. For each filtering parameter, the number of MADs was selected as 3. Cells with out-of-range values or no expression in any of the cells were removed. Spike-in datasets were filtered based on ERCC expression levels, and total counts in each cell and total features in each cell using the same procedure as UMI datasets. Filtered count matrices were normalised using *computeSumFactors* and *logNormCounts* functions of the Scran package. The per-gene variance was calculated based on a fitted mean-variance trend using *modelGeneVar* and *modelGeneVarWithSpikes* functions for droplet- and spike-based datasets, respectively. Dimension reduction (PCA) was applied and the number of principal components for further use was selected automatically using the *denoisePCA* function. The same PCA procedure was applied to the spike and droplet datasets.

**Figure 1:**
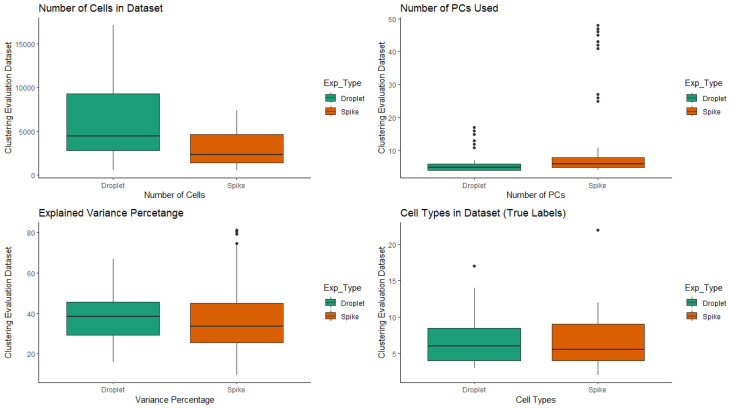
Properties of scRNA-Seq datasets.

Besides publicly available datasets, 8 additional datasets constructed by merging 2 randomly selected datasets for each class (UMI and Spike) were added to increase the range of the number of cells. The properties of the datasets (including the merged datasets) can be seen in Figure 1 and the Supplementary File (Tables S1–S4).

### 2.2. Preparation of the clustering evaluation dataset

For each dataset the clustering procedure shown in Algorithm 1 was applied. As shown, PCA is applied to 500, 1000, 1500, 2000, 2500, and 3000 highly variant genes (HVGs). SNN graphs were constructed using the number of neighbours in the range from 1 to 50 with an automatically selected number of PCs, and 3 different community detection algorithms were used for clustering, Walktrap, Louvain, and Leiden. The accuracy of clustering was evaluated using the adjusted Rand index (ARI) (Rand, 1971) based on the cell labels provided with the datasets. The output of each clustering task, which involves the ARI value, number of neighbours, number of cells, number of HVHs, clustering algorithm, number of PCs used, and percentage of variance explained using a selected number of PCs, and experiment type were stored in a data frame to be used in the model construction.

### 2.3. Training, testing, and optimisation of the random forest classifier model

The stored data frame, the clustering evaluation dataset (CED), was imported to Python to construct the random forest classifier model using Scikit-learn (Breiman, 2001; Pedregosa et al., 2012). To construct a classification model, clustering results with an ARI higher than 0.8 were labelled as “acceptable ARI”. Before model optimisation, datasets without any acceptable ARI values were removed. The dataset was split into training and test datasets in a ratio of 0.75 to 0.25, respectively. Before optimisation, the training data had 66,262 clustering results (13,256 of them were acceptable), while the test data had 22,088 clustering results (4386 of them were acceptable). The random forest (Ho, 1995) algorithm was optimised after setting the number of estimators as 2, 5, 7, 9, and 12; the minimum number of sample split as 3, 5, 8, and 10; the maximum depth as 3, 5, 8, 10, 12, and 15; the number of maximum estimators as 3, 5, 7, and 9; and class weight as 1 for nonacceptable ARI and from 1 to 3 (0.1 as step size) for acceptable ARI. The model was optimised using the grid search method (LaValle et al., 2004) and 10-fold cross-validation. The ROC AUC value was used as the scoring method for the grid search. The optimal parameters were used to construct the same model in R using the *randomForest* package in R.

### 2.4. The SUMAShiny application and Dunn index-based clustering

To extend the flexibility of the tool, 4 additional clustering methods along with SUMA were integrated into the SUMAShiny application and this provides users with alternative methods in addition to our optimised SUMA model.

In SUMAShiny, Seurat clustering (Louvain-based graph clustering) with default parameters was added as the first clustering method. As the second and third clustering methods, Louvain and Leiden algorithms with Dunn index-based clustering (Algorithm 2) were integrated to the application. In this method, the number of neighbours in the range from 1 to 50 is used to construct an SNN graph and is clustered using Louvain and Leiden algorithms. In each iteration, the Dunn index is calculated, using the *clValid* (Brock et al., 2008) library. The number of neighbours producing the clusters with the highest Dunn index is selected as the optimum k.

The fourth and final method is consensus clustering. This uses stability evidence and subsamples a proportion of features (PCs) and items (cells) from the data matrix (in this case PCA-reduced data matrix) and clusters the subsamples using a clustering method to partition the subsamples into *k* groups with a user-defined number of iterations. In SUMAShiny, the default number of iterations is set as 10, the clustering method is set as hierarchical clustering, and the maximum number of clusters is set as 25. The selection of the optimal number of clusters is automatised using the proportion of ambiguously clustered pairs (PAC) method (Şenbabaoğlu et al., 2014).

scRNA-Seq data are visualised using UMAP (McInnes et al., 2018) in Seurat. The SUMAShiny design allows visualisation of each clustering result and enables users to download clustering results as a CSV file.

## 3. Results

### 3.1. Model optimisation results

**Figure 2:**
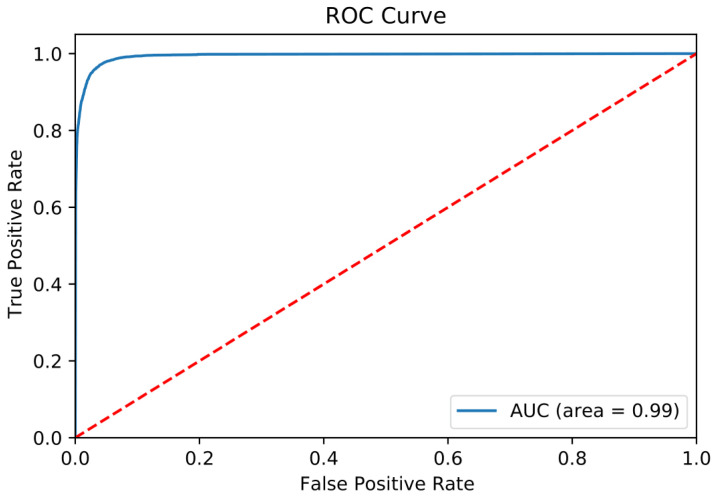
ROC curve of the test dataset.

Grid searching was used to optimise the model as a function of the number of estimators, minimum number of sample split, maximum depth, number of maximum estimators, and class weight. Receiver operating characteristic area under the curve (ROC-AUC) was maximised as an indicator of the performance of the model. The selected model had a maximum depth of 16, maximum features of 4, minimum sample split of 8, number of estimators of 16, and class weights of 1 for nonacceptable ARI and 1.8 for acceptable ARI. The test and training datasets were used to calculate the metrics to measure the prediction power of SUMA (Table 1). The results indicated that the model has high sensitivity and specificity that predicts positive (accepted ARI) and negative (nonaccepted ARI) labels while it has a very low false positive rate, which is the proportion of negatives that are incorrectly identified positives, and also a very low false discovery rate, which is the proportion of false positive results. The ROC curve of the test dataset can be seen in Figure 2.

**Figure 3:**
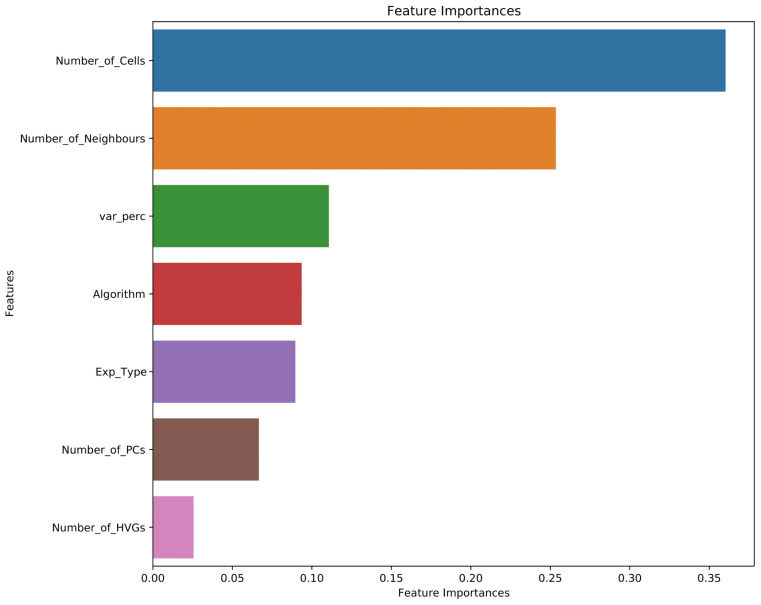
Feature importance levels of SUMA.

The feature importance levels of SUMA (Figure 3) indicated that the number of cells in the dataset is the most important feature that SUMA uses for prediction. The number of neighbours is the second most important feature, while the number of most variant genes used in clustering has the least importance.

The model was also tested with 3 independent Sincell scRNA-Seq datasets with 3 different numbers of HVGs (Table 2). In all tests, SUMA predicted the number of neighbours that give an ARI value higher than 0.9; the optimal parameters including the number of neighbours are listed in the Supplementary File (Tables S1–S4).

**Figure 4:**
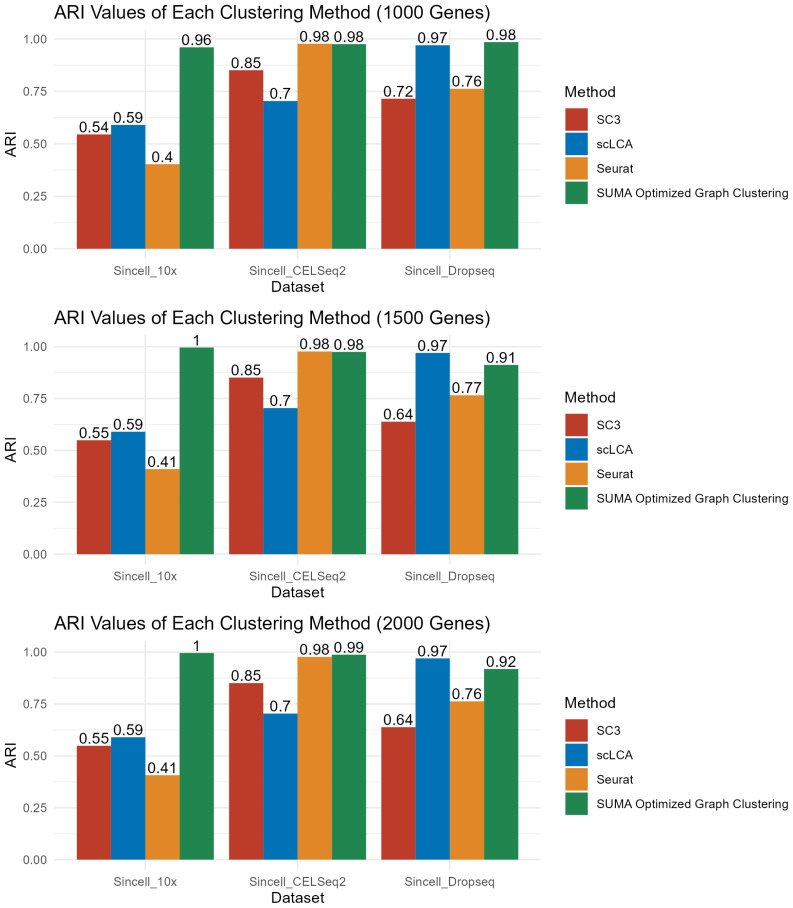
Comparison of SUMA predictions with other methods.

To demonstrate the prediction power of SUMA, the results are compared using 3 established clustering algorithms in the tools Seurat (Hao et al., 2021), SC3 (Kiselev et al., 2017), and scLCA (Cheng et al., 2019). For each method, default parameters were used. SUMA’s predicted parameters outperformed the other methods (Figure 4, Supplementary File (Tables S1–S4). The same number of principal components are used for each analysis except scLCA as scLCA applies latent cellular analysis to the count matrix rather than a reduced version of it. For SC3 clustering, the built-in function *sc3_estimate_k* is used to predict the number of clusters since SC3 uses the k-nearest neighbour algorithm for clustering. The terminal application, SUMA.py, is used to predict the parameters for optimum clustering for test and comparison analyses. The results of the comparisons (Figure 4) demonstrated that clustering with parameters optimised by SUMA outperformed the other methods in almost all cases. Irrespective of the data type, data size, or number of variant genes, clustering with the number of neighbours recommended by SUMA provided the best results, except for DropSeq data, where scLCA provided better clustering if the number of variant genes was larger than 1000.

**Figure 5:**
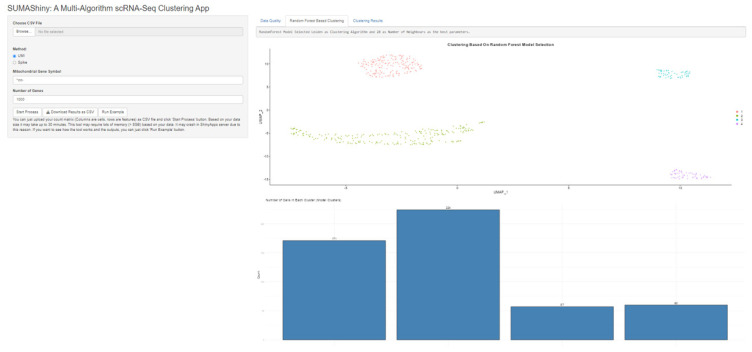
User interface of SUMAShiny.

Our application tool, SUMAShiny (Figure 5), can be used by users without any experience in scRNA analysis. The tool is completely automated, a CSV file (rows as genes, columns as cells) is uploaded as the input, and the experiment type (Spike or UMI), the number of genes, and the symbol of mitochondrial genes are specified by the user. For analysis of a dataset that has 512 cells, the desktop tool takes about 20 min on a PC with 16 threads and 48 GB of memory. SUMAShiny is connected to Shinyapps.io servers; hence, users with limited resources can run the application remotely.

## 4. Discussion

The clustering of scRNA-Seq datasets is a crucial step prior to cell type identification in the analysis pipelines. Suboptimal clustering may cause users to lose rare cell types or misidentify cell types similar to each other. The majority of the time, clustering is repeated with different parameters to produce the best results; this trial-and-error procedure requires a large number of parameter combinations to be tested to ensure selection of the best combination. Methods that use more advanced frameworks such as neural networks or LLMs may require computing power, particular operating systems, and some programming language libraries that may be challenging for nonexpert users. To address these challenges, we developed SUMA, a lightweight method to find the optimum number of neighbours in the clustering of scRNA-Seq using graph-based clustering. To extend the usability of our model, we integrated the model with widely used clustering algorithms. We believe that our model and application will help nonexperts to analyse scRNA-Seq data while providing an alternative tool for more experienced users.

SUMA is trained with a limited number of high-quality and standardised benchmarking datasets from two scRNA-Seq protocols (10X and CELSeq2). Datasets produced by 10X and CELSeq2 constitute about 90% of the scRNA-seq data in data repositories; hence, most users will be analysing data from these two protocols. Nevertheless, higher rates of false positive or false positive predictions may arise when using datasets from platforms with different experimental procedures, or datasets with high levels of noise, technical variation, or batch effect, and the accuracy of the results may be lower than that of our test results. Another limitation directly affects the web application; the Shinyapps.io server provides a limited amount of memory to users (currently 8 GB). Due to this limitation, users with large datasets should use the RShiny desktop application or the SUMA terminal application.

We integrated additional algorithms into SUMAShiny to provide users with a flexible tool that can produce clustering results based on algorithms of popular tools. Combining our machine learning model SUMA further with Dunn index-optimised algorithms and hierarchical clustering-based consensus clustering, we aimed to provide users with a variety of clustering methods with different infrastructures. Visualisation functionality in SUMAShiny allows users to directly integrate the clustering results into their analysis.

The framework used to develop SUMA can easily be adapted to new platforms and new datasets. With the increasing number of scRNA-Seq benchmarking data and the highly adaptable architecture of our tool, which allows other machine learning algorithms to be applied, SUMA and SUMAShiny will be updated regularly and its open-source code can be used by other researchers to add new features.

## Supplementary Data

**Table S1:** Data properties.

Dataset	Number of Cells	Number of Cell Types
Bladder (Spike)	1378	2
Brain Myeloid (Spike)	4455	2
Brain Nonmyeloid (Spike)	3401	7
Fat (Spike)	4865	6
Heart (Spike)	4745	9
Kidney (Spike)	519	5
Large Intestine (Spike)	3938	5
Limb Muscle (Spike)	1960	8
Liver (Spike)	714	5
Lung (Spike)	1676	11
Mammary Gland (Spike)	2405	4
Marrow (Spike)	5037	22
Pancreas (Spike)	1564	9
Skin (Spike)	2310	5
Spleen (Spike)	1697	3
Thymus (Spike)	1349	3
Tongue (Spike)	1416	2
Trachea (Spike)	1350	4
Merged Dataset 1 (Spike)	7339	12
Merged Dataset 2 (Spike)	5384	10
Merged Dataset 3 (Spike)	3119	9
Merged Dataset 4 (Spike)	4652	10
Zhengmix 4eq	3994	4
Zhengmix 4uneq	6498	4
Zhengmix 8eq	3994	8
Bladder (Droplet)	2500	4
Heart and Aorta (Droplet)	526	4
Kidney (Droplet)	2781	8
Limb Muscle (Droplet)	3909	6
Liver (Droplet)	1845	4
Lung (Droplet)	5404	13
Mammary Gland (Droplet)	4481	7
Marrow (Droplet)	3652	14
Spleen (Droplet)	9552	5
Thymus (Droplet)	1429	3
Tongue (Droplet)	7538	3
Trachea (Droplet)	11,248	5
Merged Dataset 1 (Droplet)	7262	14
Merged Dataset 2 (Droplet)	9313	17
Merged Dataset 3 (Droplet)	17,090	8
Merged Dataset 4 (Droplet)	14,033	9

**Table S2:** Clustering evaluation dataset.

Number_of_Cells	Number_of_Neighbours	Number_of_PCs	Number_of_HVGs	ARI	var_perc
Min.: 519	Min.: 1.0	Min.: 4.000	Min.: 500	Min.:0.0000	Min.: 9.48
1st Qu.: 1697	1st Qu.: 13.0	1st Qu.: 5.000	1st Qu.: 1000	1st Qu.: 0.1710	1st Qu.: 27.27
Median: 3909	Median: 25.5	Median: 6.000	Median: 1500	Median: 0.3870	Median: 36.55
Mean: 4447	Mean: 25.5	Mean: 9.022	Mean: 1749	Mean: 0.3844	Mean: 36.95
3rd Qu.: 5384	3rd Qu.: 38.0	3rd Qu.: 6.000	3rd Qu.: 2500	3rd Qu.: 0.5830	3rd Qu.: 45.52
Max.: 17,090	Max.: 50.0	Max.: 48.000	Max.: 3000	Max.: 1.0000	Max.: 81.23

**Table S3:** SUMA test parameters.

Dataset	Number of Genes (“g” parameter)	Number of Used PCs (“p” parameter)	Number of Cells (“c” parameter)	Experiment Type (“e” parameter)	Explained Variance Percentage (“v” parameter)
Sincell 10x	1000	8	902	1	63
Sincell CELSeq2	1000	42	274	0	64.01
Sincell Dropseq	1000	5	225	1	38.49
Sincell 10x	1500	5	902	1	52.54
Sincell CELSeq2	1500	41	274	0	59.09
Sincell Dropseq	1500	5	225	1	33.33
Sincell 10x	2000	5	902	1	57.47
Sincell CELSeq2	2000	40	274	0	55.44
Sincell Dropseq	2000	5	225	1	30.03

**Table S4:** Comparison with other methods.

Dataset	Number of Genes	Number of Used PCs	ARI (SUMA Selected Parameters)	Number of Clusters (Seurat)	ARI (Seurat with Default Parameters)	Number of Clusters (SC3)	ARI (SC3 with Default Parameters)	Number of Clusters (scLCA)	ARI (scLCA with Default Parameters)
Sincell 10x	1000	8	0.96	9	0.403	7	0.545	6	0.59
Sincell CELSeq2	1000	42	0.975	4	0.977	5	0.851	4	0.704
Sincell Dropseq	1000	5	0.985	5	0.7624	5	0.715	3	0.97
Sincell 10x	1500	5	0.996	9	0.41	7	0.549	6	0.59
Sincell CELSeq2	1500	41	0.975	4	0.977	5	0.851	4	0.704
Sincell Dropseq	1500	5	0.9121	5	0.766	7	0.6385	3	0.97
Sincell 10x	2000	5	0.996	9	0.407	7	0.549	6	0.59
Sincell CELSeq2	2000	40	0.9877	4	0.977	5	0.851	4	0.704
Sincell Dropseq	2000	5	0.919	5	0.763	5	0.6385	3	0.97

## Figures and Tables

**Table 1:** Prediction metrics of SUMA.

Metric	Test Data	Training Data
True Positive Rate (TPR)/Sensitivity	0.944	0.938
True Negative Rate (TNR)/Specificity	0.975	0.977
False Positive Rate (FPR)	0.024	0.023
False Discovery Rate (FDR)	0.095	0.09
ROC Area Under Curve (AUC)	0.993	0.998
Matthews Correlation Coefficient (MCC)	0.9	0.9

**Table 2:** Independent tests of SUMA with Sincell datasets.

Dataset	Number of Cells	Number of Genes	Experiment Type	Selected Algorithm	Selected k	ARI
Sincell 10x	902	1000	UMI	Leiden	39	0.96
Sincell CELSeq2	274	1000	Spike	Louvain	16	0.975
Sincell Dropseq	225	1000	UMI	Walktrap	25	0.985
Sincell 10x	902	1500	UMI	Leiden	46	0.996
Sincell CELSeq2	274	1500	Spike	Louvain	16	0.975
Sincell Dropseq	225	1500	UMI	Walktrap	23	0.91
Sincell 10x	902	2000	UMI	Leiden	39	0.996
Sincell CELSeq2	274	2000	Spike	Louvain	16	0.987
Sincell Dropseq	225	2000	UMI	Walktrap	23	0.919

**Algorithm 1:** Preparation of Clustering Evaluation Dataset

1	let X = scRNA-Seq Datasets
2	let K = Number of Neighbours
3	let P = Principal Component Analysis Result
4	let PC = Number of Principal Components Used
5	let H = Number of Highly Variable Genes
6	let A = Algorithm
7	let S = SNN Graph
8	let C = Clustering Results
9	let L = Cell Labels
10	let ARI = Adjusted Rand Index
11	let E = Experiment Type (UMI, Spike)
12	let XN = Number of Cells in scRNA-Seq Dataset
13	let VE = Percentage of Variance Explained with PC
14	for H = [500, 1000, 1500, 2000, 2500, 3000]
	for A = [Walktrap, Louvain, Leiden]
	for K = 1:50
	P, VE = *denoisePCA*(X, H)
	S = buildSNNGraph(P, K)
	C = clustering(S, Z)
	ARI(X, K, PC, H, A) = ARI(C, L)
15	ClusteringEvaluationData = [XN, H, A, K, PC, ARI, U, VE]

**Algorithm 2:** Dunn Index Optimised Clustering (Louvain and Leiden Algorithms)

1	let X = scRNA-Seq Datasets
2	let K = Number of Neighbours
3	let D = Dunn Index Value
4	let H = Number of Highly Variable Genes
5	let A = Clustering Algorithm
6	for K = 1:50
	P = *denoisePCA*(X, H)
	S = buildSNNGraph(P, K)
	C = clustering(S, A)
	D = DunnIndex(P, C)
7	selected_K = max(D)
